# Chemical Flavorants in Vaping Products Alter Neurobiology in a Sex-Dependent Manner to Promote Vaping-Related Behaviors

**DOI:** 10.1523/JNEUROSCI.0755-22.2022

**Published:** 2023-02-22

**Authors:** Skylar Y. Cooper, Clay V. Willis, Montana R. Richardson, Sean P. Hill, Sheavonnie K. Wright, Morgan B. Elmore, Zach B. Mitchell, Astrid K. González Castro, Brandon J. Henderson

**Affiliations:** Department of Biomedical Sciences, Joan C. Edwards School of Medicine, Marshall University, Huntington, West Virginia 25701

**Keywords:** dopamine, electrophysiology, microscopy, nicotinic receptor, self-administration, vaping

## Abstract

Electronic nicotine delivery systems (ENDS) are distinctly different from combustible cigarettes because of the availability of flavor options. Subjective measures have been used to demonstrate that adults and adolescents prefer flavors for various reasons; (1) they are pleasing and (2) they mask the harshness of nicotine. Despite this, there have been few investigations into the molecular interactions that connect chemical flavorants to smoking or vaping-related behaviors. Here, we investigated the effects of three chemical flavorants (hexyl acetate, ethyl acetate, and methylbutyl acetate) that are found in green apple (GA) ENDS e-liquids but are also found in other flavor categories. We used a translationally relevant vapor self-administration mouse model and observed that adult male and female mice self-administered GA flavorants in the absence of nicotine. Using α4-mCherryα6-GFP nicotinic acetylcholine receptor (nAChR) mice, we observed that mice exposed to GA flavorants exhibited a sex-specific increase (upregulation) of nAChRs that was also brain-region specific. Electrophysiology revealed that mice exposed to GA flavorants exhibited enhanced firing of ventral tegmental area dopamine neurons. Fast-scan cyclic voltammetry revealed that electrically stimulated dopamine release in the nucleus accumbens core is increased in mice that are exposed to GA flavorants. These effects were similarly observed in the medial habenula. Overall, these findings demonstrate that ENDS flavors alone change neurobiology and may promote vaping-dependent behaviors in the absence of nicotine. Furthermore, the flavorant-induced changes in neurobiology parallel those caused by nicotine, which highlights the fact that nonmenthol flavorants may contribute to or enhance nicotine reward and reinforcement.

**SIGNIFICANCE STATEMENT** The impact of flavors on vaping is a hotly debated topic; however, few investigations have examined this in a model that is relevant to vaping. Although a full understanding of the exact mechanism remains undetermined, our observations reveal that chemical flavorants in the absence of nicotine alter brain circuits relevant to vaping-related behavior. The fact that the flavorants investigated here exist in multiple flavor categories of vaping products highlights the fact that a multitude of flavored vaping products may pose a risk toward vaping-dependent behaviors even without the impact of nicotine. Furthermore, as the neurobiological changes have an impact on neurons of the reward system, there exists the possibility that nonmenthol flavorants may enhance nicotine reward and reinforcement.

## Introduction

Nicotine is the primary addictive component of all tobacco products, including electronic nicotine delivery systems (ENDS; Stolerman and Jarvis, 1995; Cullen et al., 2018, 2019), through its ability to alter dopamine (DA) neurotransmission (Sulzer, 2011; Faure et al., 2014). Although the impact of nicotine on the mesocorticolimbic system is well characterized, previous studies revealed that the actions of nicotine in the medial habenula (MHb) control behaviors related to withdrawal, anxiety, and aversion (Fowler et al., 2011; Zhao-Shea et al., 2013; Shih et al., 2014; Harrington et al., 2016; Pang et al., 2016). In one study, knocking out α5 nicotinic acetylcholine receptor (nAChR) subunits in the MHb promoted a dramatic increase in nicotine intake (Fowler et al., 2011). This highlights the fact that the MHb is a critical component of the neural mechanisms that govern smoking-related and vaping-related behaviors. Although much attention has been paid to α5 in the MHb, this region also contains α6-containing and α3-containing nAChRs (Shih et al., 2014). Of note, α6-containing nAChRs are among the nAChR subtypes that exhibit the highest potency to nicotine and nicotinic ligands (Tumkosit et al., 2006; Kuryatov and Lindstrom, 2011).

A key contributor to ENDS use is the availability of chemical flavorants that are otherwise banned in combustible products (Schneller et al., 2019). We previously showed that menthol and green apple (GA) flavorants alter reward-related behavior, nAChR upregulation, nAChR assembly, and midbrain DA neuron firing (Henderson et al., 2016, 2018; Avelar et al., 2019; Cooper et al., 2020). We observed that GA flavorants, in the absence of nicotine, can cause reward-related behaviors while altering DA neuron function (Avelar et al., 2019; Cooper et al., 2020). We also showed that GA flavorants enhance ventral tegmental area (VTA) DA neuron firing (farnesol; Avelar et al., 2019) or increase the sensitivity of VTA DA neurons to nicotine (farnesene; Cooper et al., 2020) to alter reward-related behavior. The major weakness of these previous studies is the fact that they used an injection-based assay (conditioned place preference) with low translational value to vaping-related behaviors.

Using mouse e-Vape self-administration (EVSA), we also showed that GA flavorants hexyl acetate (HA), ethyl acetate (EA), and methylbutyl acetate (MBA) promote self-administration in the absence of nicotine (Cooper et al., 2021). The shift away from our previously investigated GA flavorants (farnesol, farnesene) was made in light of previously determined common components of GA ENDS products (Tierney et al., 2016). It is also important to clarify that ethyl acetate and methylbutyl acetate are found in other flavor categories (vanilla-flavored and fruit-flavored e-liquids). Accordingly, the observation that these flavorants can modulate behavior implies that the impact goes beyond just GA-flavored ENDS. Although we have shown that mice self-administer GA flavorants at a level similar to nicotine, we have yet to investigate the contribution of the individual flavorants or how they alter neurobiology and neurophysiology.

In the present investigation, we used a vaping-relevant vapor inhalation model to examine the impact that popular GA e-liquids (mixture of hexyl acetate, ethyl acetate, and methylbutyl acetate, commonly at a ratio of 3:1:1, respectively; Tierney et al., 2016) exert on behaviors associated with ENDS use. With the above consideration in mind, we refer to this mixture as “GA-mix”; but acknowledge the fact that chemicals such as ethyl acetate and methylbutyl acetate appear in other flavor profiles. Here, we investigated individual GA flavorants in their role to alter vaping-related behaviors in the absence of nicotine. We observed that GA flavorants alter midbrain DA and habenular neuron function through changes in nAChR density and stoichiometry. Although we show the neurobiological changes are sex specific, we also show the net result on VTA DA neuron function is the same between sexes and results in enhanced DA release in the nucleus accumbens (NAc) core. Together, these results show GA flavorants alter nAChRs in the mesolimbic pathway, either directly through the VTA or indirectly through changes in the medial MHb, which suggests that GA flavors reinforce continued vaping-related behavior.

## Materials and Methods

### Mice

All experiments were conducted in accordance with the *Guidelines for the Care and Use of Laboratory Animals* provided by the National Institutes of Health. Protocols were approved by the Institutional Animal Care and Use Committee at Marshall University. Mice were group housed on a standard 12 h light/dark cycle at 22°C and given food and water *ad libitum*. For microscopy and behavioral assays, we used α4-mCherryα6-GFP mice (Henderson et al., 2017), originated from a C57BL/6J strain, that are genetically modified to contain α4-mCherry and/or α6-GFP nAChR subunits (Henderson et al., 2017; Avelar et al., 2019; Akers et al., 2020). These mice are deposited at the Mutant Mouse Resource & Research Centers (MMRRC) and available for general use (MMRRC 068051-MU). On postnatal day 21, tail biopsies were taken for genotyping analysis by PCR (Transnetyx). Only mice that were transgenic for α6-GFP and homozygous for α4-mCherry were used in confocal assays (see below), with the exception of α6-GFP and α4-mCherry mice used for NFRET controls. Following behavioral assays (see below), mouse brains that were homozygous for α4-mCherry and transgenic for α6-GFP were used in confocal microscopy assays. For electrophysiology assays, we used α6-GFP mice (discussed below). All mice were adults (3–5 months old). Both male and female mice were used, and numbers of each are detailed below for specific experiments and given in detail in corresponding figures.

### Drugs and e-liquid composition

Hexyl acetate (catalog #A0032) and methylbutyl acetate (catalog #A1076) were obtained from TCI Chemicals, ethyl acetate was obtained from Chem-Impex International (catalog #00757), nicotine salt (ditartrate dihydrate) was obtained from Acros Organics (catalog #AC415660500), (-)-menthol was obtained from Alfa Aesar (catalog #A10474), dihydro-β-erythroidine (DhβE) hydrobromide was purchased from ApexBio (catalog #B7030), propylene glycol was obtained from Tedia (catalog# PR1494-065), and vegetable glycerin was obtained from J.T. Baker (catalog #2143-01). All e-liquids were mixed with the vehicle, propylene glycol and vegetable glycerin (PGVG; 50:50 ratio), at a final concentration of 15 mg/ml for flavor (GA-mix, hexyl acetate, ethyl acetate, or methylbutyl acetate) and 6 mg/ml nicotine (menthol plus nicotine e-liquids only). For GA-mix we used hexyl acetate, ethyl acetate, and methylbutyl acetate (3:1:1, respectively) following analytical investigations into commercial e-liquids (Tierney et al., 2016; Omaiye et al., 2019).

### e-Vape self-administration assays

EVSA assays were conducted using a commercial vapor self-administration setup (La Jolla Alcohol Research; https://www.ljari.tech; Cooper et al., 2021; Henderson and Cooper, 2021). Operant vapor self-administration was conducted in airtight chambers with interior dimensions of 21 cm long × 19 cm wide × 12.5 cm high (https://www.ljari.tech). Two standard Med Associates nose pokes (containing yellow cue lights) were mounted above the floor on the backside walls of the chamber. Noncontingent (passive) vapor exposure was also conducted in airtight chambers with interior dimensions of 21 cm long × 19 cm wide × 12.5 cm high (https://www.ljari.tech); however, these chambers lacked nose poke ports. All chambers were housed in a dark Plexiglas enclosure that minimized extraneous light and noise (operant chambers in one enclosure and passive chambers in another). Airflow was vacuum controlled by an electric pump that allowed air flow at 1 L/min. The air outlet was located at the top back corner of the right wall of the chamber and connected through tubing to a HEPA-Cap filter (catalog #WHA-2609T, Midland Scientific). The vapor port was located in the front of the chamber, and the e-liquid solutions were contained in Uwell Crown IV atomizer tanks (0.40Ω dual coil; Shenzhen Uwell Technology), which were activated by a custom e-cigarette mod box (La Jolla Alcohol Research). Vapor delivery settings were controlled by an e-Vape custom controller at 400 °F and 65 W (La Jolla Alcohol Research). At these settings, each 3 s vapor delivery consumes ∼0.09 ml of e-liquid as ∼33 deliveries will consume ∼3 ml.

**Figure 1.**
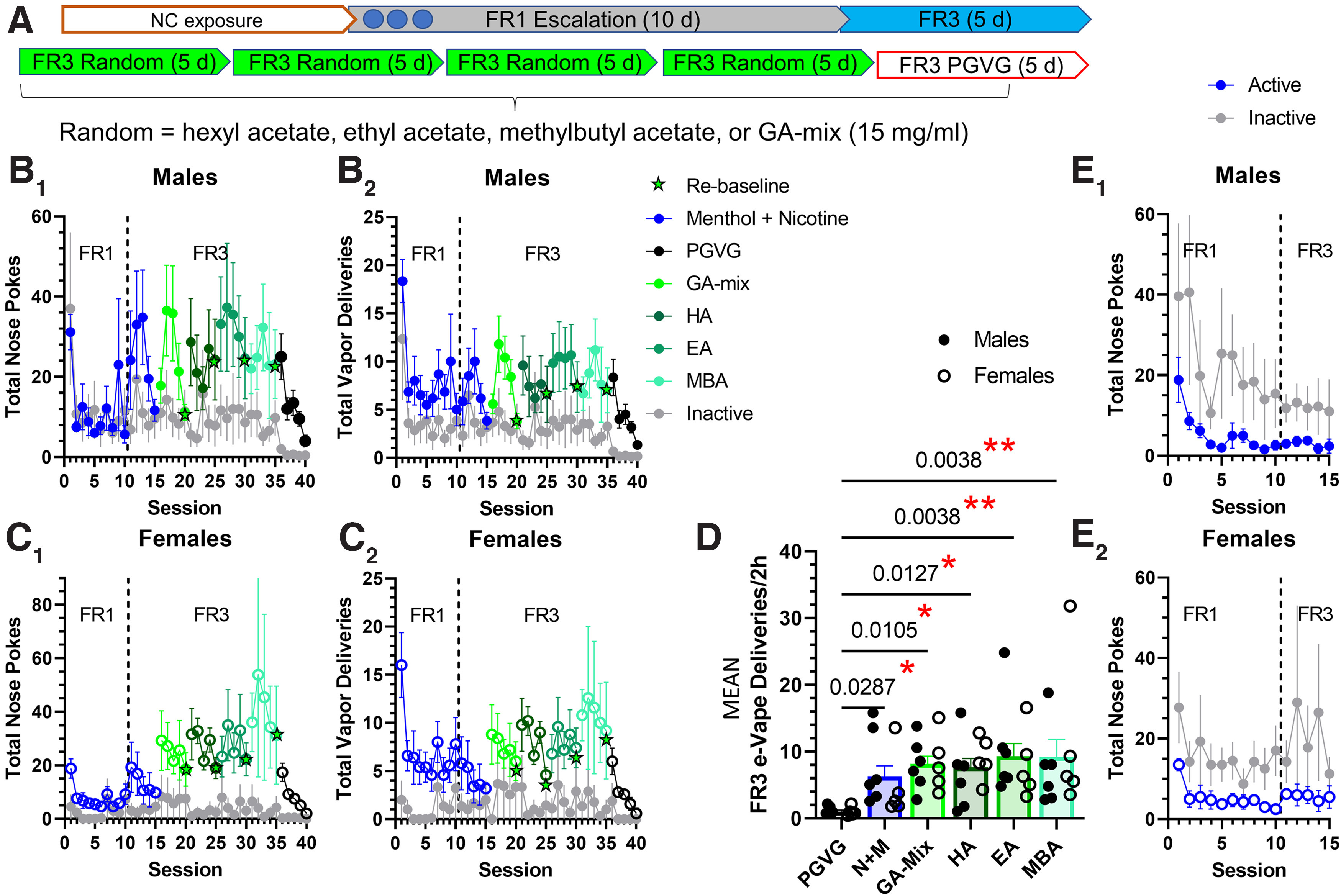
GA e-liquids promote vapor self-administration in male and female mice. ***A***, Mice were acclimated to vapor exposure during five daily 2 h noncontingent (NC) exposure sessions, then acquired vapor self-administration during 10 daily 2 h FR1 sessions followed by five daily 2 h FR3 sessions following previously established methods (Cooper et al., 2021; Henderson and Cooper, 2021). Following acquisition, mice were randomly assigned GA e-liquids until each mouse was exposed to all conditions. ***B_1_–C_2_***, Nose pokes and earned EVSA deliveries for male (***B_1_*_,_*_2_***) and female (***C_1_*_,_*_2_***) mice (6 males and 5 females). ***D***, Mean EVSA-earned deliveries for all responding on a FR3 schedule for males (closed circles) and females (open circles). Sexes were combined (*n* = 12). ***E_1_*_,_*_2_***, Active and inactive nose pokes for mice that failed to acquire a 2:1 active/inactive ratio (*n* = 5 males, 7 females). Data are mean ± SEM. Data were analyzed by two-way ANOVA or one-way ANOVA. *p < 0.05; **p < 0.01.

During their photophase, adult male and female mice began vapor exposure acclimation on a Monday for 5 d with 15 mg/ml menthol plus 6 mg/ml nicotine salt (3 s puff, 25 deliveries/2 h). This e-liquid composition provides the most robust and consistent self-administration responding in mice following acclimation (Cooper et al., 2021). This 5 d acclimation protocol differs from our previous 3 d protocol (Cooper et al., 2021), resulting in a higher success rate for mouse acquisition of self-administration behavior in pilot studies (2:1 active/inactive ratio). Following acclimation, mice were transitioned to a fixed-ratio 1 (FR1) self-administration schedule on a Monday for 10 daily 2 h sessions, with a weekend abstinence period. Mice were singly placed into airtight operant chambers that contained two nose pokes (one active and one inactive). Nose pokes in the active hole of the operant chambers resulted in a 3 s delivery of vaporized e-liquids through the vapor entrance port with a 30 s timeout. During the timeout, a yellow cue light remained on in the active nose poke hole. Inactive nose pokes were recorded with no consequences. After session 10, mice were transitioned to a FR3 schedule and maintained on nicotine plus menthol for five sessions. Maintaining an average 2:1 active/inactive ratio during this period was a criteria for progressing to the next stage of EVSA (five males and seven females, were excluded; six males and five females acquired a 2:1 active/inactive distinguishing ratio). Active and inactive nose pokes for male and female mice that were excluded are shown in Figure 1*E_1,2_*.

Following EVSA training and exclusions, mice were used in a within-subject, counterbalanced design to test the following e-liquids: 15 mg/ml hexyl acetate, ethyl acetate, or methylbutyl acetate and 15 mg/ml GA-mix (hexyl acetate, ethyl acetate, methylbutyl acetate; 3:1:1 ratio; Fig. 1*A*). Mice were maintained on a given e-liquid for 4 consecutive days (starting on a Monday) to reach stable responding and were rebaselined to 15 mg/ml menthol plus 6 mg/ml nicotine on day 5 (Friday). Following FR3 with these respective chemical flavorant assignments, mice were assigned to 50:50 PGVG (vehicle) to examine extinction-related behaviors. The mean of the four sessions each week of FR3 was used to compare reinforcement-related behavior between e-liquid assignments. For the final assignment to PGVG, we used the last three sessions to calculate mean eVape deliveries attributed to PGVG because of the fact that mice may exhibit higher responding because of extinction-related drug seeking.

A separate cohort of male and female mice were used to pair EVSA responding to GA flavorants with changes in neurophysiology. Male (*n* = 6) and female (*n* = 5) mice were acclimated to vapor delivery for 5 d noncontingently and assigned GA-mix in EVSA chambers for 10 sessions on an FR1 schedule and then 5 sessions on a FR3 schedule. To model physiological changes at all levels of vaping-related behavior, no mice were excluded in this study. Within 30 min of the last FR3 session, individual mice were anesthetized, and brains were extracted for electrophysiology (described below).

### Confocal imaging of mouse brain slices

**Figure 2.**
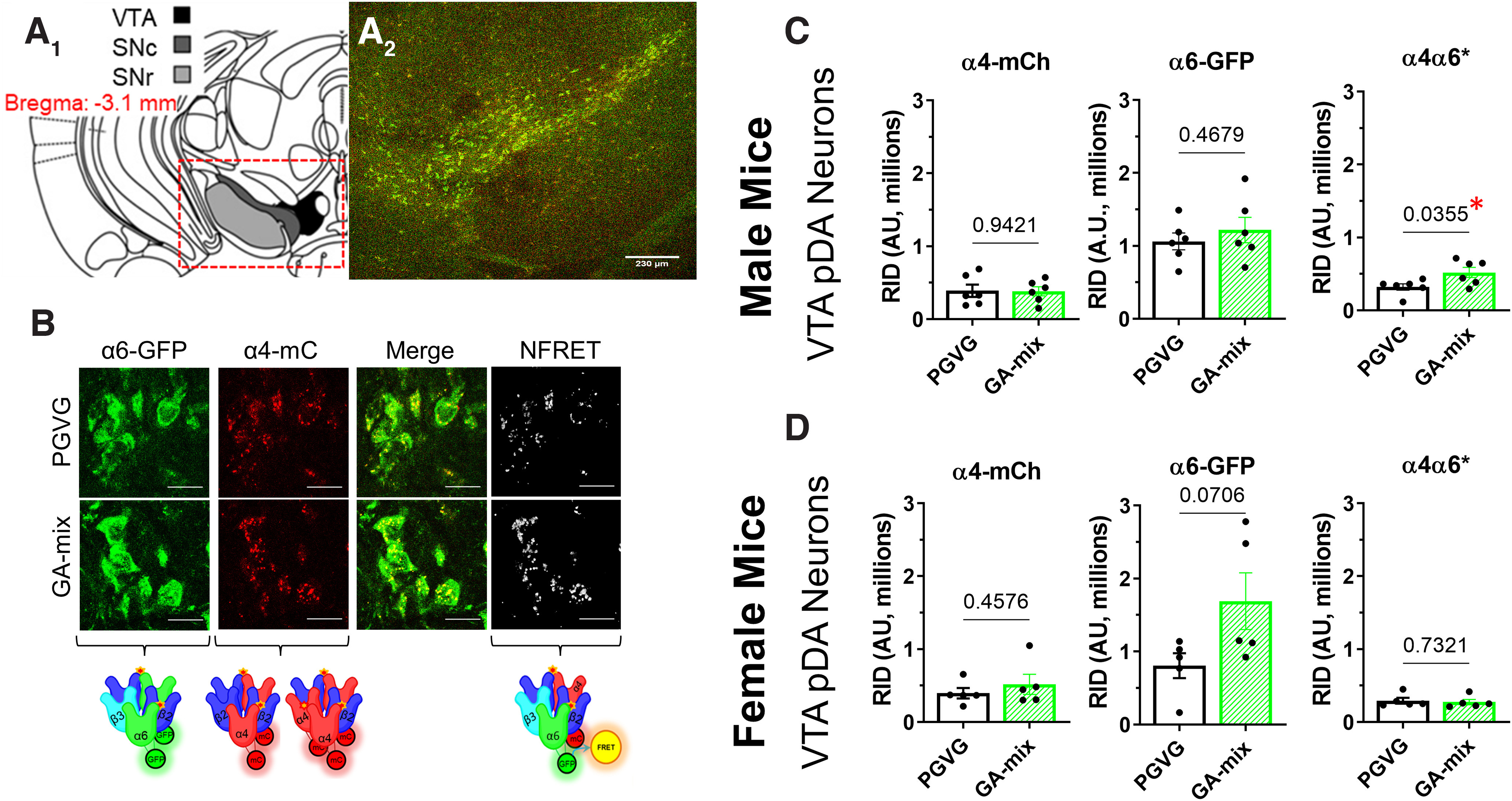
GA-mix upregulates α4α6* nAChRs in a sex-dependent manner. ***A_1_***, Schematic of target mouse brain region (bregma, −3.1 mm). ***A_2_***, Sample 10× image of a mouse coronal brain section at target bregma. ***B***, Sample images of PGVG-treated and GA-treated VTA dopamine neurons. Scale bar, 20 µm. ***C***, ***D***, RID of α4*, α6*, and α4α6* nAChRs on VTA dopamine neurons in male (*n* = 12, ***C***) and female (*n* = 10, ***D***) mice. Dots indicate the RID values from individual mice. Data are mean ± SEM; two-way ANOVA with *post hoc* Bonferroni followed by unpaired *t* test.

Although we have previously shown that mice will self-administer GA e-liquids (Cooper et al., 2021), we had not yet examined whether vaporized delivery of GA flavorants alters nAChR density in the brain regions relevant to reward or reinforcement. Because of the fact that our mice undergoing self-administration were used in a within-subjects design, we used a separate cohort of mice exposed to only 15 mg/ml GA-mix or PGVG for 10 d. Using previously validated methods (Henderson et al., 2017; Avelar et al., 2019; Cooper et al., 2020), we examined α4β2 (α4-mCherry), α6β2* (α6-GFP), and α4α6β2* nAChRs on VTA DA (α6-GFP+) neurons in α4-mCherryα6-GFP mice (Fig. 2).

The α4-mCherryα6-GFP mice were passively exposed to 15 mg/ml GA-mix or PGVG for 10 daily 2 h sessions at a rate of 25 deliveries per session (3 s puff). Following the final vapor exposure session on Friday, mice were transported to our standard laboratory and were killed with CO_2_ within 30 min of the end of their session. After cardiac perfusion with 10 ml ice-cold saline to reduce autofluorescence in the mCherry emission range, brains were removed, flash frozen with acetone and dry ice, and then stored at −80°C. Brains were coronally sectioned (20 µm) using a cryostat, mounted with VECTASHIELD (catalog #H-1000, Vector Laboratories), and coverslipped. We targeted bregma −3.1 mm (anterior–posterior limits of −2.9 to −3.3 mm; Allen Brain Atlas, mouse.brain-map.org) for consistent sections of the midbrain, as well as bregma −1.8 mm (anterior–posterior limits of −1.5 to −2.0 mm; Allen Brain Atlas) for habenula and hippocampal regions.

A Leica SP5 TCSII confocal microscope was used to excite α6-GFP and α4-mCherry at 488 and 561 nm, respectively; 20× images with a 10× digital zoom (for the midbrain) and 20× images with a 5× digital zoom (for habenula/dentate gyrus) were collected for the quantitative measurements of α4-mCherry and α6-GFP neurons. Similar to previously used methods to investigate nAChR upregulation (Henderson et al., 2014, 2016, 2017), raw integrated density (RID), which provides a measure for changes in fluorescent intensity and area, was used for quantitative measures. This provides a benefit over analyzing mean intensity as nAChR upregulation in neurons involves translocation of receptors away from the soma. Normalized Förster Resonance Energy Transfer (NFRET) was calculated using the PixFRET ImageJ plug-in to identify α4α6* (the asterisk indicates other subunits may be present) nAChRs in the VTA (Henderson et al., 2017; Avelar et al., 2019; Akers et al., 2020). We (Akers et al., 2020) and others (Mackey et al., 2012) have shown that α6* nAChRs exhibit a >95% overlap with tyrosine hydroxylase and is a suitable marker for dopamine neurons in both the VTA and substantia nigra pars compacta. Accordingly, we used α6-GFP fluorescence as a marker for putative DA (pDA) neurons.

**Figure 3.**
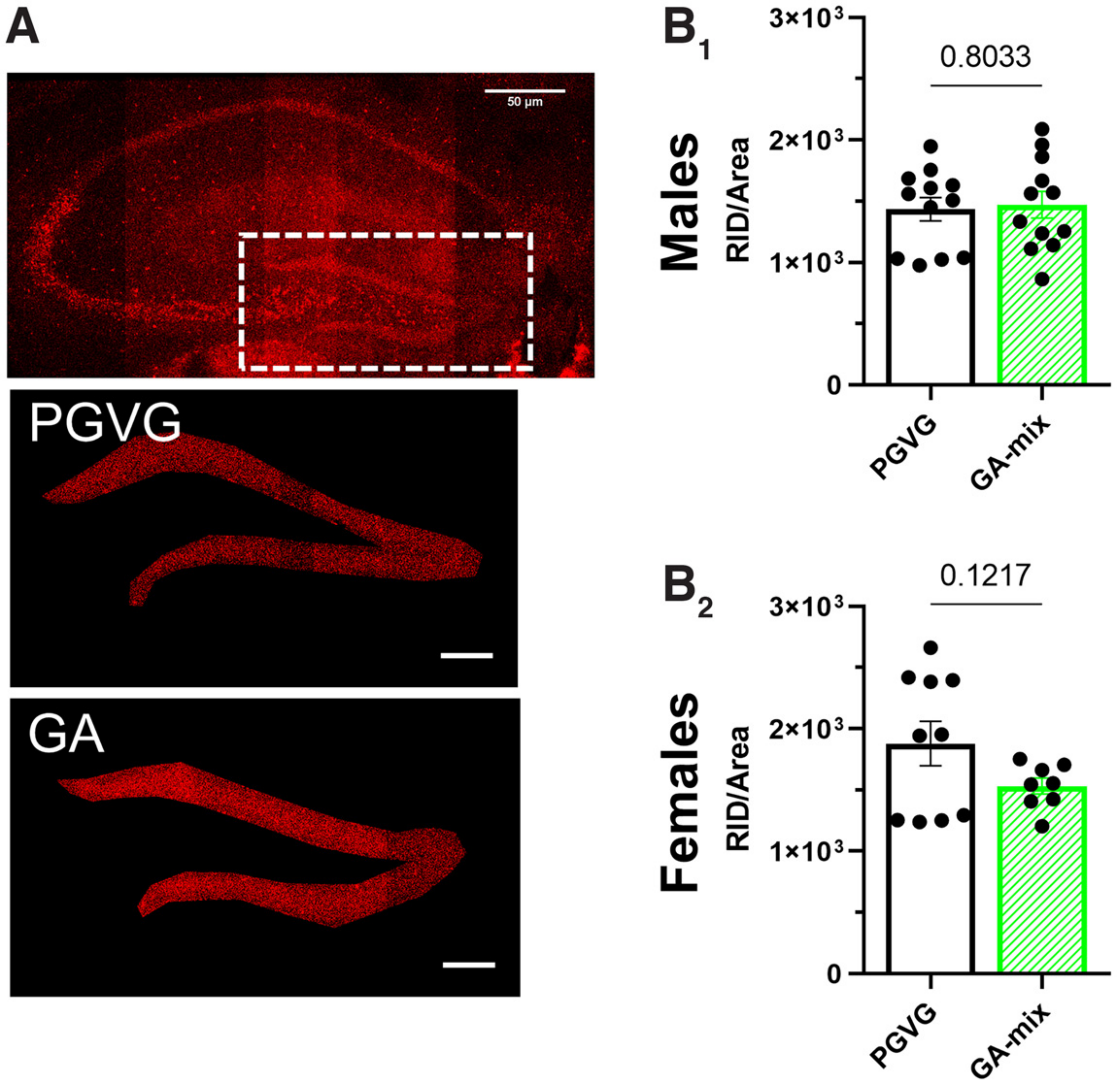
GA-mix does not alter nAChR density in the dentate gyrus. ***A***, Sample 10× image of a mouse coronal brain section at target bregma (−1.8 mm). ***B_1_*_,_*_2_***, RID of a4* nAChRs on dentate gyrus neurons in PGVG-treated and GA-treated male (***B_1_***; *n* = 12 for PGVG and GA groups) and female (***B_2_***; *n* = 10 and 8 for PGVG and GA groups, respectively) mice. All data are mean ± SEM; two-way ANOVA with *post hoc* Bonferroni test followed by unpaired *t* test. Dots (***B_1_*_,_*_2_***) indicate the RID values from each hippocampal region of individual mice.

All experimenters were blind to drug treatment until all data analysis was completed. Approximately 30–60 VTA dopamine neurons and the entirety of the medial habenula (MHb) and hippocampus (bilaterally) were imaged. Data from these images were averaged to provide RID values for each mouse. A total of 27 mice were used in confocal assays (*n* provided in Figs. 2, 3; see Fig. 6).

### Patch-clamp electrophysiology

**Figure 4.**
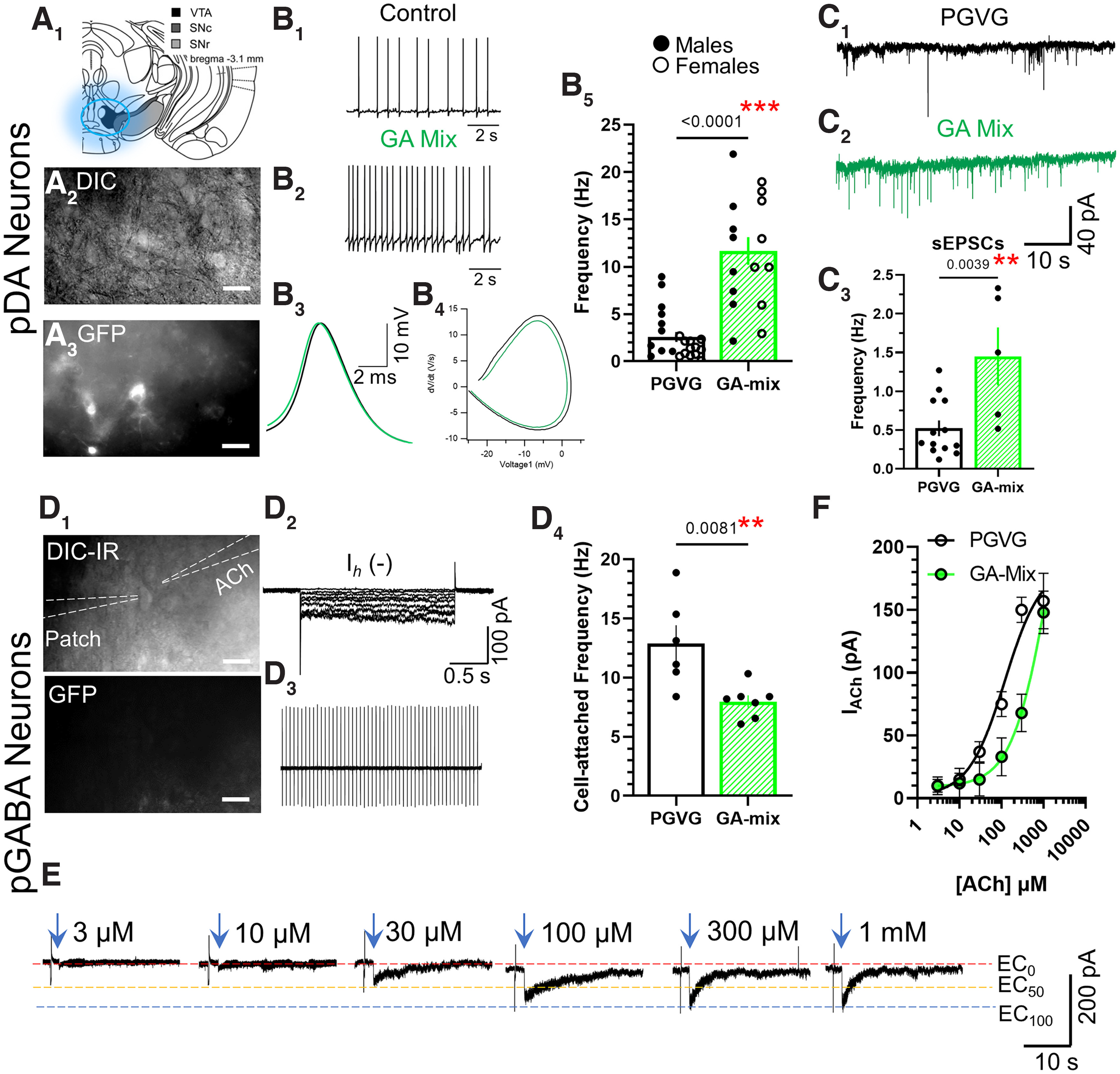
GA-mix alters VTA DA and GABA neuron firing. ***A_1–3_***, Representative images of VTA dopamine neurons identified by α6-GFP fluorescence. ***B_1–4_***, Representative waveforms of VTA DA neuron firing from mice exposed to PGVG or GA-mix. ***B_5_***, Mean data of VTA DA neuron firing frequency for PGVG-treated male (black dots; *n* = 18 neurons, 4 mice) and female (open dots; *n* = 10 neurons, 3 mice) mice and GA-mix-treated male (black dots; *n* = 8 neurons, 5 mice) and female (open dots; *n* = 10 neurons, 3 mice) mice. ***C_1–3_***, Representative waveforms (***C_1_*_,_*_2_***) and mean data (***C_3_***) of VTA DA neuron sEPSCs recorded from mice exposed to PGVG (*n* = 13 neurons, 4 male mice) or GA-mix (*n* = 5 neurons, 5 male mice). ***D_1_*_,_*_2_***, Representative placement (***D_1_***) of patch and puffer orientation to target VTA GABA neurons and putative GABA neurons were GFP(−) and I*_h_*(−) (***D_2_***). ***D_3_*_,_*_4_***, VTA GABA neuron firing frequency in mice exposed to PGVG or GA-mix. ***E***, ***F***, Representative waveforms (***E***) and mean data (***F***) of increasing concentrations of ACh applied to VTA GABA neurons from mice exposed to PGVG and GA-mix. All data are mean ± SEM; two-way ANOVA with *post hoc* Bonferroni test followed by unpaired *t* tests. Data are mean of 3–6 VTA GABA neurons (***F***). Dots within bars represent the values from individual cells within the designated treatment group. Scale bars: 20 µm.

Although our neurobiology assays facilitate the examination of changes in nAChR density, our microscopy methods do not provide information regarding function. Therefore, we used patch-clamp electrophysiology to examine changes in function following exposure to GA-mix. Using brain slices from 3- to 5-month-old male and female α6-GFP mice, we identified pDA neurons in the VTA because of the selective expression of α6* nAChRs in pDA neurons (Mackey et al., 2012; Akers et al., 2020). Following previous work detailing the presence of α6* nAChRs on medial VTA glutamate neurons (Yan et al., 2018), we restricted our recordings to the lateral VTA to increase our chance of accurately identifying pDA neurons. In addition to examining changes in VTA pDA neurons, we also examined VTA GABA neurons. VTA putative GABA (pGABA) neurons were identified in the lateral VTA by absence of GFP fluorescence and absence of I*_h_* (Fig. 4*D*_1–3_).

Following passive vapor exposure to 15 mg/ml GA-mix or PGVG for 10 daily, 2 h sessions (3 s puff, 25 deliveries/session), mice were anesthetized with CO_2_ and then cardiac perfusion was performed using ice-cold NMDG-based artificial CSF (NMDG-ACSF) saturated with 95%/5% O_2_/CO_2_ (carbogen) containing the following (in mm): 93 NMDG, 2.5 KCl, 1.2 NaH_2_PO_4_, 10 MgSO_4_, 0.4 CaCl_2_, 30 NaHCO_3_, 5 Na-ascorbate, 3 Na-pyruvate, 2 thiourea, and 25 glucose. Brains were placed in agarose for slicing with a Compresstome VF-300-OZ (Precisionary Instruments). Coronal brain sections (250 µm) were cut into cold carbogenated NMDG-ACSF to obtain slices containing the VTA (target bregma, −3.1 mm; anterior–posterior limits of −2.9 to −3.3 mm; Allen Brain Atlas), MHb (bregma, −1.8 mm; anterior–posterior limits of −1.5 to −2.0 mm; Allen Brain Atlas), or NAc (see below, Fast-scan cyclic voltammetry) and were then allowed to recover at 32°C in carbogenated NMDG-ACSF for 12–15 min. Following this, slices were transferred to standard ACSF containing the following (mm): 125 NaCl, 2.5 KCl, 1.2 NaH_2_PO_4_, 1.2 MgCl_2_, 2.4 CaCl_2_, 26 NaHCO_3_, and 11 glucose for 1 h at 32°C. One hour later, slices were transferred to the recording chamber and superfused with carbogenated ACSF (1.5–2.0 ml/min) at 32°C.

Neurons were visualized with an Axio Examiner A1 (Zeiss) equipped with an Axiocam 702 mono. Patch-clamp techniques were used to record electrophysiological signals with an Integrated Patch-Clamp Amplifier (Sutter Instrument) using previously described methods (Avelar et al., 2019; Akers et al., 2020; Cooper et al., 2020). Patch electrodes had resistances of 4–10 MΩ when filled with intrapipette solution containing the following (in mm): 135 K gluconate, 5 KCl, 5 EGTA, 0.5 CaCl_2_, 10 HEPES, 2 Mg-ATP, and 0.1 GTP. Recordings were sampled at ≥10 kHz. The junction potential between patch pipette and bath solutions was nulled just before gigaseal formation. Series resistance was monitored without compensation throughout experiments using SutterPatch software. The recording sessions for neurons were terminated if the series resistance changed by >20%. In whole-cell recordings, recordings were made after 5 min to provide sufficient time for interchange of intrapipette solution with intracellular components.

**Figure 5.**
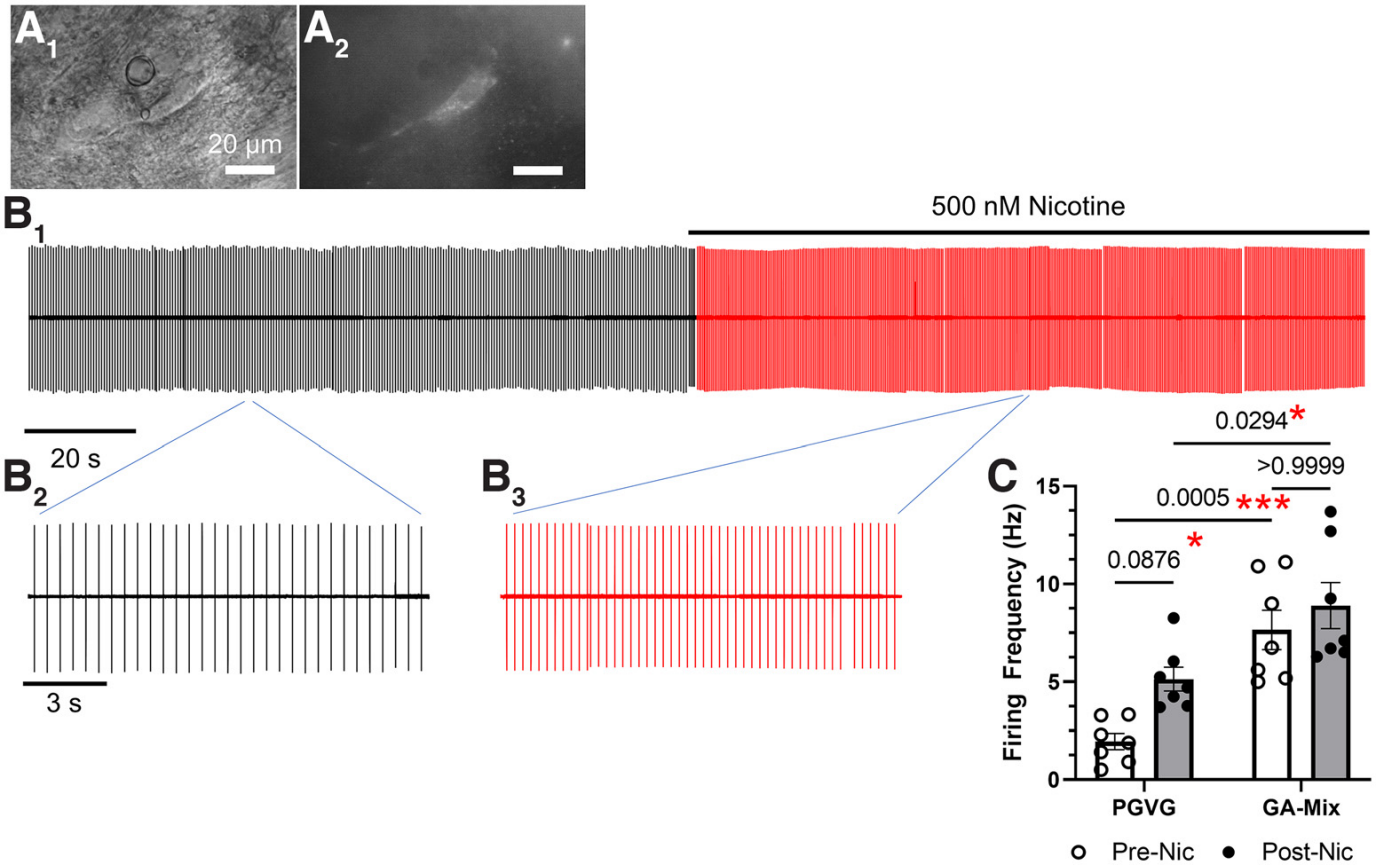
Acute nicotine does not enhance VTA DA neuron firing in mice treated long-term with GA-mix. ***A_1_*_,_*_2_***, Representative VTA pDA neuron in differential interference contrast and GFP imaging modes. ***B_1_***, Four-minute trace of cell-attached recording before (black) and during (red) application of 500 nm nicotine. ***B_2_*_,_*_3_***, Highlighted waveforms of cell-attached firing prenicotine and during nicotine. ***C***, Firing frequency data of mice exposed to PGVG or GA-mix prenicotine and postnicotine application. Nic, Nicotine. Data are mean ± SEM; two-way ANOVA with *post hoc* Bonferroni test. Dots within bars represent the values from individual cells within the designated treatment group. (For PGVG treated, 7 cells were obtained from 3 individual mice; for GA-mix treated, 7 cells were obtained from 3 individual mice.)

**Figure 6.**
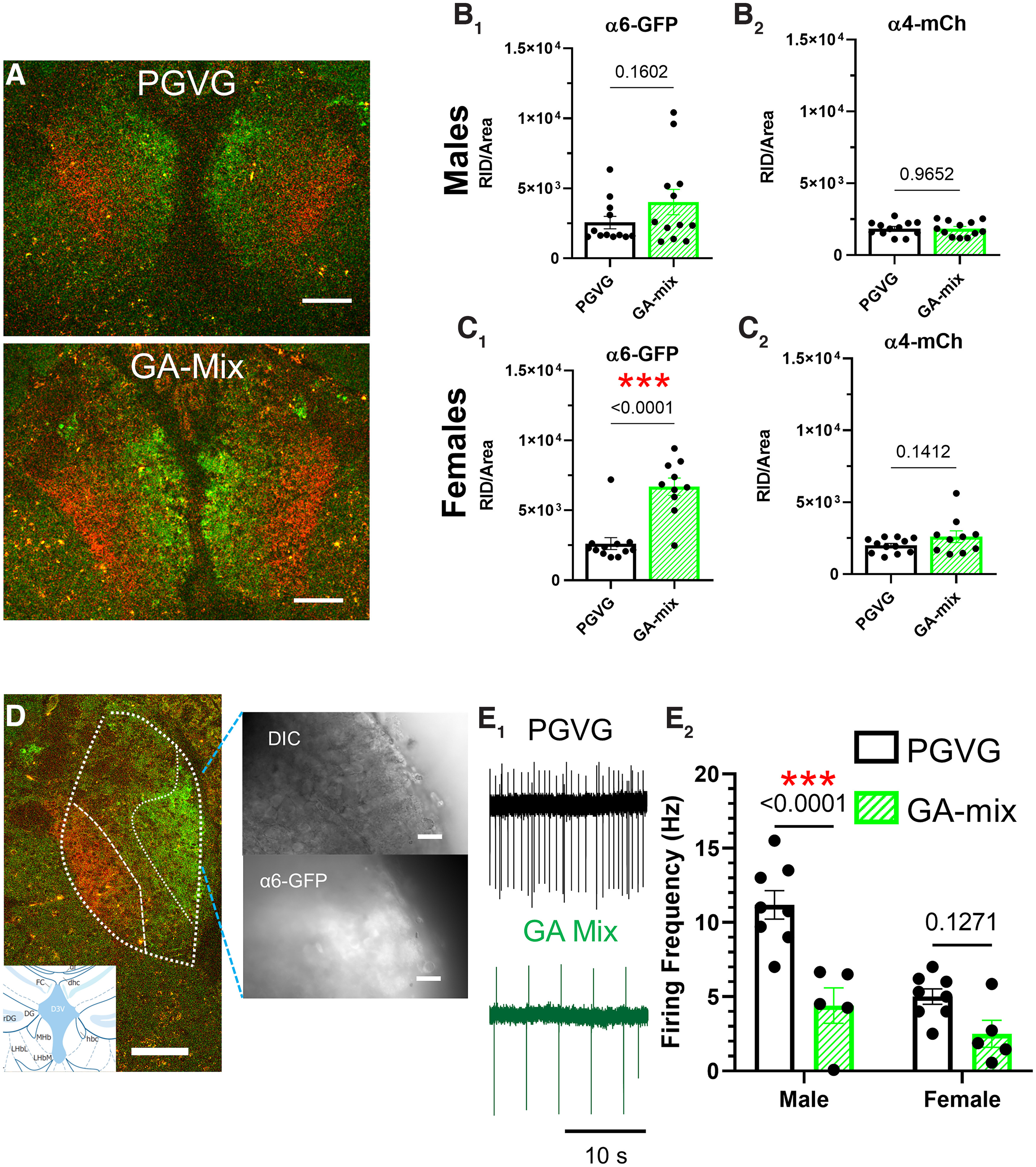
GA-mix alters nAChR density and functionality of neurons in the medial habenula. ***A***, Sample 10× image of an α4-mCherryα6-GFP mouse coronal brain section at target bregma (−1.8 mm) from PGVG-treated and GA-mix-treated mice. ***B_1_–C_2_***, RID of α4* (***B_2_***, ***C_2_***) and α6* (***B_1_***, ***C_1_***) nAChRs on medial habenula neurons in PGVG-treated and GA-treated female (*n* = 12 PGVG and 10 GA-mix) and male (*n* = 12 PGVG and 12 GA-mix) mice. ***D***, Representative images of slices prepared from α4-mCherryα6-GFP mice for electrophysiology. ***E_1_***, Representative cell-attached recordings of medial MHb neurons from slices prepared from PGVG-treated or GA-mix-treated mice. ***E_2_***, Mean ± SEM, data of cell-attached firing frequencies (males, *n* = 8 neurons, 3 mice; 5 neurons, 3 mice) for PGVG and GA-mix, respectively (females, *n* = 8 neurons, 3 mice; 5 neurons, 3 mice for PGVG and GA-mix, respectively). Data were analyzed by two-way ANOVAs with a *post hoc* Bonferroni (***B_1_*_,_*_2_***, ***C_1_*_,_*_2_***), followed by unpaired *t* tests., Data were analyzed by two-way ANOVAs with a *post hoc* Bonferroni (***E_2_***). Dots within bars indicate the values from individual mice (microscopy) or individual cells (electrophysiology). Scale bars, 20 µm.

For the recordings of spontaneous EPSCs (sEPSCs), perfusion of ACSF was switched to an ACSF solution containing 100 μm picrotoxin (catalog #124-87-8, Sigma-Aldrich) to block GABAA receptors. After 5 min, DA neurons in the VTA were voltage clamped at −65 mV to record sEPSCs. To isolate ACh-induced nAChR currents in VTA GABA neuron recordings, standard ASCF (see above) was supplemented with 0.5 μm atropine. For VTA electrophysiology assays, a total of seven PGVG-treated (four males, three females) and eight GA-mix-treated (five males, three females) mice were used. For each mouse recordings from 2–3 neurons were used (*n* provided in Fig. 4). For VTA electrophysiology that used 0.5 μm nicotine applications, three PGVG-treated and three GA-mix-treated mice were used (*n* provided in Fig. 5). For MHb electrophysiology assays, a total of six PGVG-treated (three males, three females) and six GA-mix-treated (three males, three females) mice were used (*n* provided in Fig. 6). For each mouse, recordings from 1–3 neurons were used.

For the recordings of intrinsic excitability following EVSA assays, mice were allowed to acquire EVSA behavior using GA-mix for 10 FR1 sessions and then assigned five FR3 sessions (*n* = 6 males, 5 females). Following this, brains were extracted and used in electrophysiology assays using identical brain slice preparations as described above. Intrinsic excitability in α6-GFP-positive pDA neurons and α6-GFP-positive medial MHb neurons was assessed by using a stepped-current injection protocol in current-clamp mode (5 pA steps, −20 pA to 70 pA). Rheobase was determined from the threshold current sufficient to elicit an action potential. Electrophysiological data were correlated to the mean FR3 active nose pokes from all five of the FR3 sessions of the individual mouse. For these electrophysiology assays, VTA recordings used 11 neurons from seven mice (three male, four females), and medial MHb neurons used eight neurons from six mice (three males, three females). Of the mice used in the corresponding EVSA assays (as above, six males and five females), some mice were used in recordings for both the VTA and MHb.

### Fast-scan cyclic voltammetry 

**Figure 7.**
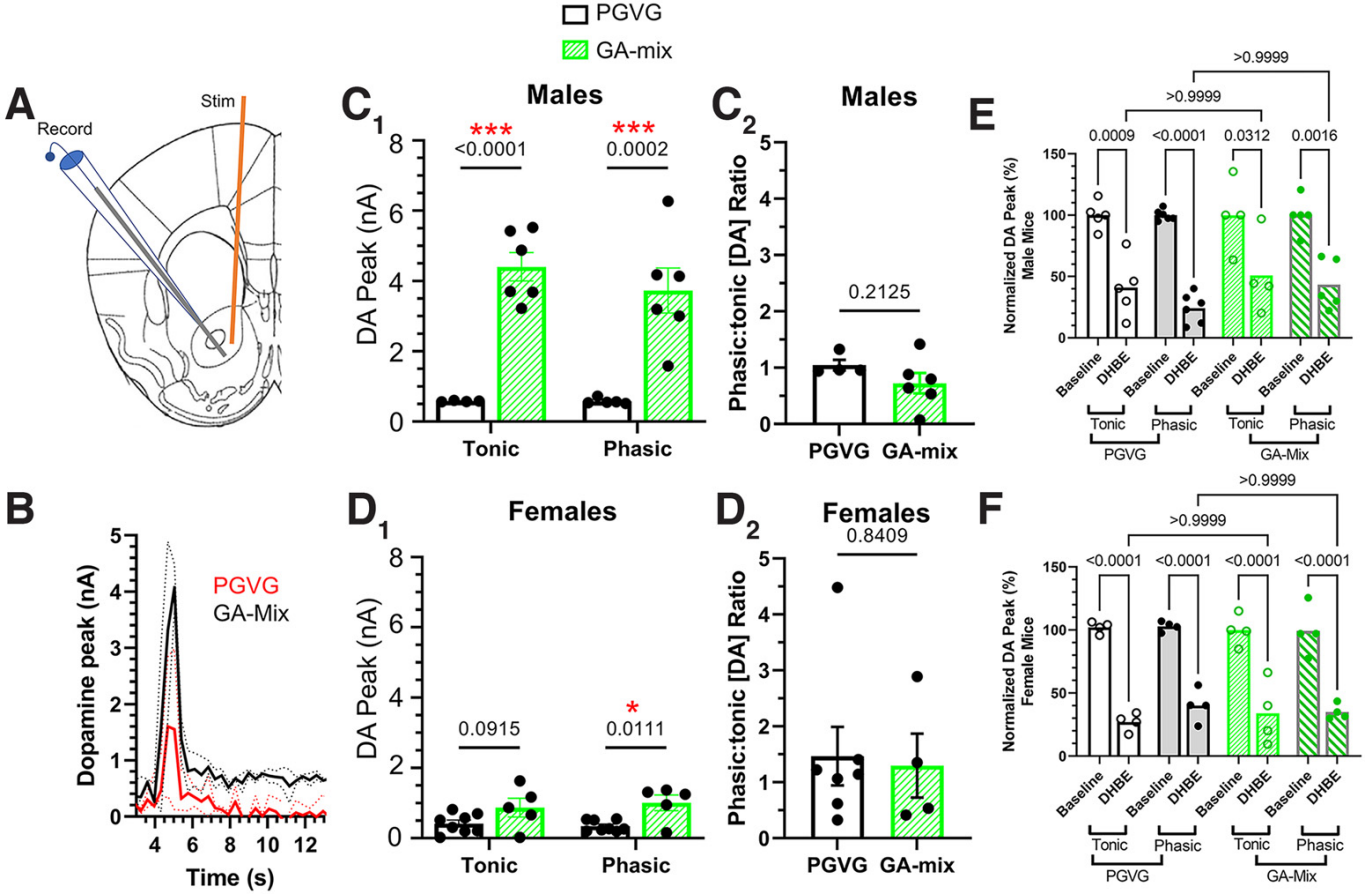
GA-mix enhances DA release through the mesolimbic pathway. ***A***, Schematic of stimulating and recording electrode placement at the NAc core (bregma, +1.0 mm). ***B***, Representative waveforms of PGVG-treated and GA-treated mice following tonic (5 Hz) and phasic (60 Hz) stimulus trains. ***C_1_***, ***D_1_***, Mean DA peak response in male and female mice treated with PGVG or GA-mix. ***C_2_***, ***D_2_***, Phasic/tonic ratio of PGVG-treated and GA-mix-treated mice. ***E***, ***F***, Nucleus accumbens core DA release pre- and post-DhβE (10 μm) application. All data are mean ± SEM. Males, *n* = 5 and 6 for PGVG and GA-mix, respectively; females, *n* = 8 and 5 for PGVG and GA-mix, respectively (***C***, ***D***). Males, *n* = 6 and 4 for PGVG and GA-mix, respectively; females, *n* = 5 and 4 for PGVG and GA-mix, respectively (***E***, ***F***). Two-way ANOVA with *post hoc* Bonferroni (***C_1_***, ***D_1_***, ***E***, ***F***) or unpaired *t* test (***C_2_***, ***D_2_***).

Brain slices including the NAc core (target bregma, +1.0 mm; anterior–posterior limits of +1.4 to +0.7 mm; Allen Brain Atlas) were collected for fast-scan cyclic voltammetry (FSCV) using methods identical to those in the electrophysiology assays. After recovery, slices were transferred to the recording chamber, and a carbon-fiber microelectrode was lowered to the NAc core. A 2 kHz triangular waveform (−0.4 V to +1.0 V and back to −0.4 V, at a rate of 400 V/s) was applied at 20 Hz (Integrated Patch Amplifier, Sutter Instrument). Dopamine release was stimulated (350 µA, Master-9) with a bipolar electrode (Plastics One), placed ∼250 µm from the recording electrode (100–250 µm away). Tonic-like stimulation was elicited with a five-pulse train at 5 Hz (0.2 ms interpulse interval, 1 s total stimulation time). Phasic-like stimulation was elicited with five-pulse trains at 60 Hz (16 ms interpulse intervals, 83 ms total stimulation time). Electrical stimulation was delivered at 2 min interstimulus intervals to avoid signal rundown. Electrodes were calibrated using 0, 0.01, 0.1, 1, and 10 μm dopamine standards. A total of 24 mice were used (11 males; five PGVG treated, six GA-mix treated) and 13 females (eight PGVG treated, five GA-mix treated); *n* provided in Fig. 7). To minimize the number of mice used, some of the above-listed mice were the same mice used in VTA electrophysiological assays (seven PGVG-treated mice; four males, three females) and eight GA-mix treated mice; five males, three females). Here, slices containing the NAc were collected immediately after the sections that included the VTA. The remaining mice of the 24 total, were separate cohorts that were exposed to PGVG or GA-mix.

To examine changes in nAChR-mediated DA release, a separate cohort of mice was exposed to PGVG or GA-mix using identical conditions as those above. Identical five-pulse 5 Hz and 60 Hz recordings were recorded in the absence and presence of 10 μm DhβE, a β2-containing nAChR antagonist. Here, DhβE was dissolved into ACSF at a concentration of 10 μm. FSCV recordings were completed in standard ACSF, and after a stable baseline was determined the perfusion was switched to the ACSF that included DhβE. After 3 min, FSCV recordings were completed in the presence of DhβE (for males, *n* = 6 and 4 for PGVG and GA-mix, respectively; for females, *n* = 5 and 4 for PGVG and GA-mix, respectively).

### Neuroblastoma-2a cell culture and transient transfections

We next examined nAChR stoichiometry in transiently transfected neuroblastoma-2a cells using previously validated NFRET methods (Henderson et al., 2016, 2017; Avelar et al., 2019; Cooper et al., 2020). Mouse neuroblastoma-2a cells were cultured in MEM with 5% fetal bovine serum, 100 IU/ml penicillin, and 100 µg/ml streptomycin. Cells were plated by adding 80,000 cells to 35 mm glass-bottom imaging dishes (MatTek) and cultured in a humidified incubator (37°C, 95% air, 5% CO_2_). For NFRET assays, cells were transfected with 500 ng of each nAChR subunit cDNA plasmid (α4-mCherryα4-GFPβ2-WT for α4β2, α6-mCherryβ2-WTβ3-YFP for α6β2β3, α4-mCherryα6-GFPβ2-WTβ3-WT for α4α6β2β3, and α3-GFPα5-mCherryβ4-WT for α3α5β4 nAChR subtypes). Following plating procedures, plasmid DNA was mixed with 50 µl of Opti-MEM and P3000 at 2 µl/µg. Lipofectamine 3000 was separately added to another 50 µl of Opti-MEM. After 5 min at 24°C, the two solutions were combined and incubated at 24°C for 25 min. Plated cells then received the mixed solution, with an additional 2 ml of Opti-MEM, and were incubated for 24 h. The following day, 500 nm filter-sterilized hexyl acetate, ethyl acetate, or methylbutyl acetate was added after replacing the Opti-MEM with standard culture medium. Each experiment included a sham control (no drug addition). This concentration (500 nm) was chosen based on perceived pharmacologically relevant concentrations of tobacco flavorants (Henderson et al., 2016, 2017, 2018). Twenty-four hours after drug addition, cells were fixed with 4% PFA for 20 min, washed twice with 1× extracellular solution, mounted with VECTASHIELD (catalog #H-1000, Vector Laboratories) and coverslipped, then imaged on a confocal microscope (*n* > 30 cells per condition).

### Calcium 6 assay (FlexStation)

The calcium 6 procedure was used with minor modifications of a previously published procedure using Fluo-4 (González-Cestari et al., 2009; Henderson et al., 2010). For this calcium accumulation assay, HEK293T cells transiently expressing α4β2 nAChRs were plated at a density of 1.5–2.0 × 10^5^ cells per well in clear 96-well culture plates previously coated with poly-l-ornithine. On the day of the experiment, cells were washed with 100 µl extracellular solution (Henderson et al., 2010) and incubated in the dark for 1 h at 24°C with 50% Calcium 6 No-Wash dye (Molecular Devices). The plates were then placed into a fluid handling integrated fluorescence plate reader (FlexStation 3, Molecular Devices), and fluorescence was read at excitation of 485 nm and emission of 525 nm from the bottom of the plate with changes in fluorescence monitored at ∼1.5 s intervals. For assessment of agonist activity, 100 μm concentrations of GA flavorants (hexyl acetate, ethyl acetate, or methylbutyl acetate) were delivered to α4β2 nAChRs, and the fluorescent response was monitored for 60 s. As a control, 300 μm nicotine was used to stimulate maximal α4β2 nAChR activity. For antagonist assessment, increasing concentrations of GA flavorants (1, 10, 100, 300, 1000 μm) were added with 300 μm nicotine and a previously determined GA flavorant, farnesol (Avelar et al., 2019), which acts as an antagonist and was used as a control for this experiment.

### Statistical analyses

**Figure 8.**
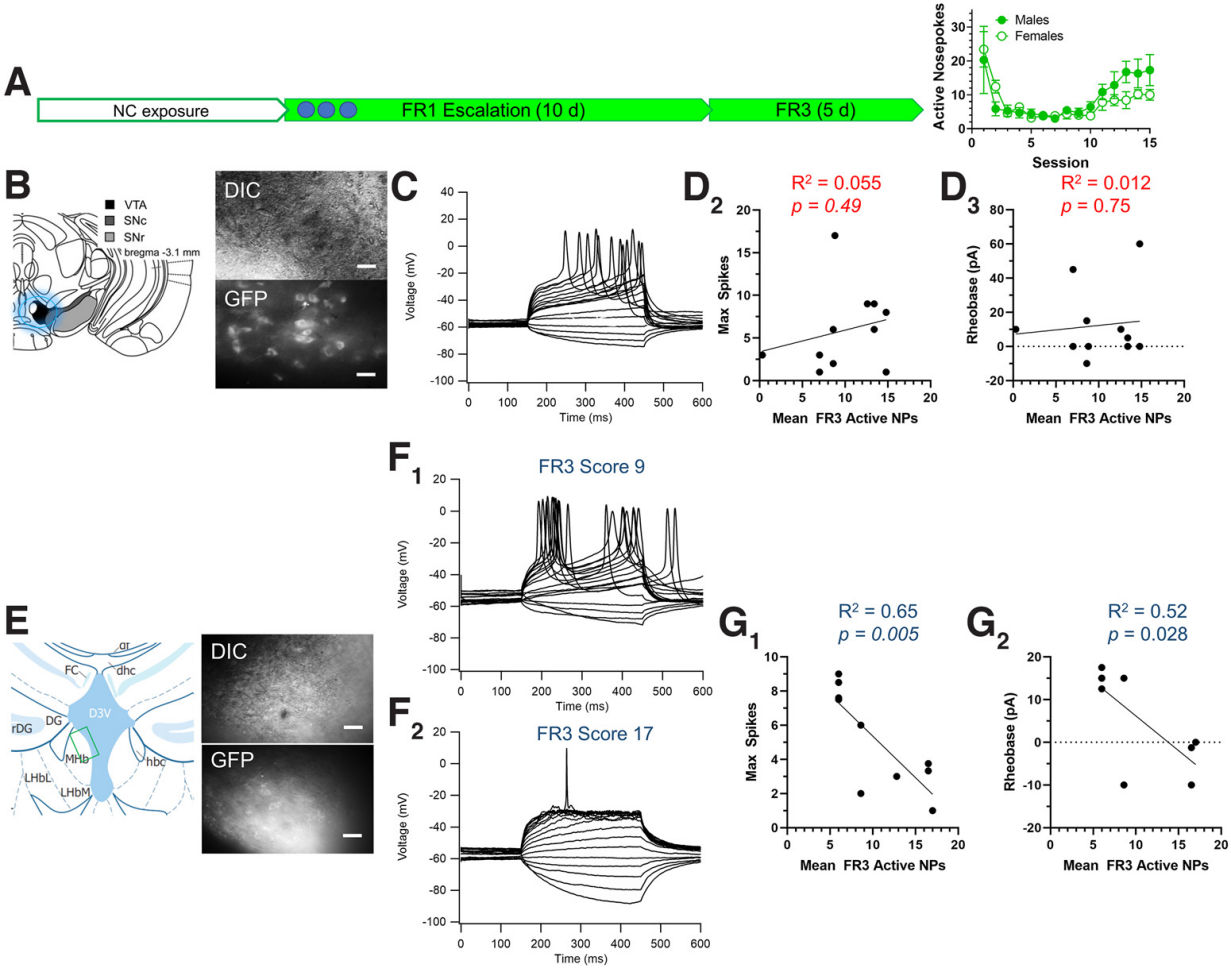
Reinforcement-related behavior associated with GA-mix inversely correlates to α6* MHb neuronal excitability and not VTA pDA neuron excitability. ***A***, Schematic of EVSA paradigm using only GA-mix (left) and mean active nose pokes (right) of male (*n* = 6) and female (*n* = 5) mice. ***B***, Schematic and representative images of VTA pDA neurons in differential interference contrast (DIC) and GFP imaging modes. ***C***, Representative stepped-current voltage recordings from α6-GFP-containing VTA pDA neurons. ***D_1_*_,_*_2_***, Correlation of mean FR3 responding (active nose pokes) to VTA pDA neuron rheobase (***D_1_***) and maximum spike number during current steps (***D_2_***). ***E***, Schematic and representative images of DIC and GFP imaging modes of medial MHb neurons. ***F_1_*_,_*_2_***, Representative stepped-current voltage recordings from α6-GFP-containing VTA pDA neurons at two different FR3 scores. ***G_1_*_,_*_2_***, Correlation of mean FR3 responding (active nose pokes) to medial MHb neuron rheobase (***G_1_***) and maximum spike number during current steps (***G_2_***). Scale bars: 20 µm.

All results are presented as mean ± SEM, and all statistical analyses were performed using GraphPad Prism 9 software. FR3 e-Vape responding and N2a NFRET assays were analyzed with a one-way ANOVA (Figs. 1*D*, 8). Sex-specific, e-liquid-specific, or time-specific differences were determined through a mixed-effects, two-way, ANOVA with sex, e-liquid, and/or time as factors (Figs. 1-7). Significant effects following ANOVAs were followed with a *post hoc* Bonferroni test. Figures 2, 3, 4, 6, and 7, *C2* and *D2*, were analyzed through a Student’s unpaired *t* test following two-way ANOVA analysis. Figure 5 was analyzed through a Student’s paired *t* test following two-way ANOVA analysis. Grubb’s outlier test was used to detect outliers; however, none were found. Full statistical data are provided in Results below and in the figure legends.

## Results

### Male and female mice self-administer vaporized GA flavorants

Using a previously described paradigm of EVSA (Henderson and Cooper, 2021), male and female adult mice escalated their nose poke behavior during their transition from FR1 to FR3 (Fig. 1*B1*,*C1*). Male and female mice also were observed to earn similar numbers of EVSA deliveries during chemical flavorant sessions when compared with nicotine plus menthol (Fig. 1*B2*,*C2*). To examine reinforcement-related behavior of the groups, we calculated the mean of the last four sessions for each e-liquid assignment (Fig. 1*D*). Upon analysis by two-way ANOVA, we detected a significant difference with e-liquid assignment (*F*_(5,54)_ = 3.37, *p =* 0.010), however, we failed to detect a difference among sexes [two-way ANOVA, *F*_(1,54)_ = 0.012, *p =* 0.91 (sex factor); *F*_(5,54)_ = 0.57, *p =* 0.91 (interaction); *F*_(1,54)_ = 3.37, *p =* 0.010 (drug factor)]. With no sex differences detected, males and females were combined in the analysis of FR3 EVSA deliveries. Both males and females exhibited significant FR3 responding to nicotine plus menthol, GA-mix, and individual flavorants compared with PGVG [*F*_(5,60)_ = 3.5, *p =* 0.007; one-way ANOVA; Fig. 1*D*; *post hoc* Bonferroni analyses, nicotine plus menthol (N+M), *p =* 0.029; GA-mix, *p =* 0.011; HA, *p =* 0.013; EA, *p =* 0.004; and MBA, *p =* 0.004]. Finally, all mice exhibited extinction-related behaviors when assigned PGVG (vehicle) within five sessions (Fig. 1*B–D*).

### Vaporized exposure to GA flavorants induces nAChR upregulation in the VTA

Because of previously identified sex-specific effects (Avelar et al., 2019), we examined nAChR upregulation on VTA pDA neurons in a sex-specific manner. In both males and females, mice exposed to GA-mix exhibited no change in α4* (*p =* 0.94 and 0.46 for males and females, respectively, unpaired *t* test) or α6* (*p =* 0.47 and 0.07 for males and females, respectively, unpaired *t* test) nAChR density on VTA pDA neurons compared with PGVG mice (Fig. 2*C*,*D*). We note that females exhibited an approximately twofold increase in α6-GFP RID; however, this was not statistically significant (*p =* 0.07, Fig. 2*D*). In males only, we noted a significant increase in α4α6* nAChRs on VTA pDA neurons following exposure to GA-mix (*p =* 0.04, Fig. 2*C*). We did not observe any change in α4α6* nAChR density in female mice following exposure to GA-mix (*p =* 0.73, Fig. 2*D*).

Nicotine has been shown to upregulate α4β2 nAChRs in the dentate gyrus (Nashmi et al., 2007). In the same mice that were used to examine changes in VTA pDA neurons, we cut slices containing the hippocampus to examine how exposure to chemical flavorants altered α4β2 nAChRs in the dentate gyrus. Exposure to GA-mix did not contribute to a change in α4β2 nAChR density in the dentate gyrus in either males (*p =* 0.80) or females (*p =* 0.12; Fig. 3).

### GA flavorants alter pDA neuron function in the VTA

Here, we exposed mice to PGVG or GA-mix identical to the paradigm used in our microscopy assays. In VTA pDA neurons, we observed that both male and female mice exposed to GA-mix exhibited a significant increase in baseline firing frequency [two-way ANOVA, *F*_(1,34)_ = 42.0, *p <* 0.0001 (drug factor); *F*_(1,34)_ = 0.48, *p =* 0.49 (sex factor); *F*_(1,34)_ = 1.38, *p =* 0.25 (interaction); Fig. 4*B_1–5_*; *post hoc* Bonferroni analyses: PGVG vs GA-mix (males), *p <* 0.0001; PGVG vs GA-mix (females), *p <* 0.0001]. This change in firing frequency was not accompanied by any change in action potential spike waveform (action potential amplitude, width, or afterhyperpolarization; Fig. 4*B3*_,_*4*). We also noted a significant increase in the frequency of sEPSCs in VTA pDA neurons, recorded in the presence of the GABA_A_ antagonist picrotoxin (Fig. 4*C_1–3_*, *p =* 0.004, unpaired *t* test; sexes combined). We detected no difference in sEPSC amplitude (data not shown).

GA-mix-exposed mice exhibited a decrease in VTA GABA firing frequency compared with PGVG-exposed mice (Fig. 4*D4*; *p =* 0.008). We applied increasing concentrations of the endogenous nAChR agonist, acetylcholine (ACh), in the presence of atropine to block muscarinic AChRs (Fig. 4*E*) to VTA GABA neurons and noted a change in ACh potency with neurons from GA-mix-exposed mice compared with PGVG-exposed mice (Fig. 4*F*). VTA GABA neurons do not contain α6 nAChR subunits and are composed of α4β2 nAChRs, which have been shown to exhibit low- and high-sensitivity agonist states (Nelson et al., 2003; Moroni et al., 2006; Tapia et al., 2007; Srinivasan et al., 2011). Accordingly, this change in ACh potency suggests a potential change in α4β2 nAChR stoichiometry.

As a follow-up to investigations into GA-mix-induced changes in VTA pDA neuron firing frequencies, we examined how VTA pDA neurons responded to acute applications of nicotine at smoking/vaping-relevant concentrations following exposure to PGVG or GA-mix. Here, we recorded cell-attached firing frequencies until a stable response was observed (2 min) and then switched perfusion to an ACSF solution that contained 500 nm nicotine (Fig. 5). Using a two-way ANOVA, we observed a significant effect of drug assignment (PGVG vs GA-mix, *F*_(1,14)_ = 30.5, *p =* 0.0001) and acute nicotine (*F*_(1,24)_ = 6.69, *p =* 0.02). However, we did not detect a significant effect of interaction (*F*_(1,24)_ = 1.29, *p =* 0.27). We observed a nicotine-induced increase in VTA pDA neuron firing frequency during acute nicotine exposure with control (PGVG treated) conditions (Fig. 5*C*; *p =* 0.03, *post hoc* Bonferroni) that was similar to previous investigations (Nashmi et al., 2007; Avelar et al., 2019). In brain slices prepared from male mice exposed to GA-mix, we noted elevated baseline firing (*p =* 0.0005, *post hoc* Bonferroni) when comparing firing frequency of GA-mix (prenicotine) to PGVG (prenicotine) that was similar to our previous observations (Fig. 4*B5*). However, we did not observe a significant increase in firing frequency during application of 500 nm nicotine with GA-mix conditions (Fig. 5*C*; *p =* 0.63). Despite this, we detected a significant difference in postnicotine VTA pDA firing frequency when comparing GA-mix treatment and PGVG treatment (*p =* 0.03, *post hoc* Bonferroni, Fig. 5*C*).

### GA flavorants alter nAChR upregulation in the medial habenula in a sex-dependent manner

The MHb is a key mediator of mechanisms related to nicotine, including aversion, anxiety, and withdrawal (Fowler et al., 2011; Shih et al., 2014; Harrington et al., 2016; Pang et al., 2016). Accordingly, we examined α6* and α4* nAChRs in the medial and lateral MHb regions, respectively (Fig. 6*A*). On analysis by two-way ANOVA, we detected a significant sex difference among α4* nAChR density (*F*_(1,42)_ = 4.25, *p =* 0.046) and α6 nAChR density (*F*_(1,11)_ = 5.93, *p =* 0.03). We also detected a significant difference with e-liquid assignment (*F*_(1,11)_ = 34.32, *p =* 0.0001) and sex times e-liquid interaction (*F*_(1,9)_ = 7.07, *p =* 0.026) among α6* nAChR density only. Following exposure to GA-mix, we observed an increase in α6* nAChR density in the medial MHb (mMHb) in only female mice (Fig. 6*C1*; *p <* 0.0001, unpaired *t* test). We failed to detect a change in MHb α6* nAChRs in male mice (*p =* 0.16) or in MHb α4* nAChRs in male and female mice (males, *p =* 0.97; females, *p =* 0.14; Fig. 6*B*,*C*).

Next, we examined GA-induced changes in mMHb neuron function as identified by α6-GFP expression in slices prepared from α4-mCherryα6-GFP mice (Fig. 6*D*). Here, a two-way ANOVA detected a significant effect of drug (GA-mix) treatment that was sex dependent (*F*_(1,22)_ = 26.2, *p <* 0.0001, drug factor; *F*_(1,22)_ = 19.8, *p =* 0.0002, sex factor; *F*_(1,22)_ = 5.54, *p =* 0.028, interaction). On the basis of sex differences, we observed the baseline firing frequency of neurons recorded from male and female PGVG-treated mice to differ by a factor of ∼2 (11.2 and 4.8 Hz, respectively; Fig. 6*E2*). Neurons that were α6-GFP(+) in the mMHb were observed to exhibit about a twofold, but nonsignificant, decrease in firing frequency in female mice following exposure to GA-mix (Fig. 6*E2*; *p =* 0.13, *post hoc* Bonferroni). Despite observing no change in MHb nAChR upregulation in male mice (Fig. 6*B1*), we noted a significant decrease in mMHb neuron firing frequency following exposure to GA-mix (*p <* 0.0001; Fig. 6*E2*; *post hoc* Bonferroni). The change in firing frequency in neurons from male mice highlights the fact that the nonsignificant increase in mMHb α6* nAChRs (57.4% increase; Fig. 6*B1*) may be biologically significant even if it is not statistically significant. We detected no change in sEPSCs (data not shown). Given this sex-specific difference in MHb baseline firing, we also note that we did not see a difference in VTA pDA neuron firing between sexes (Fig. 4*B5*).

### GA flavorants enhance DA release in the NAc core

We next stimulated NAc core DA release using pulse trains to emulate tonic and phasic firing (five pulses at 5 Hz and five pulses at 60 Hz, respectively; Fig. 7). At all stimulation phases we observed that PGVG-treated male and female mice resulted in DA peaks of ∼0.5 nA (Fig. 7*C1*,*D1*). Male mice exposed to GA-mix exhibited enhanced DA release at both tonic and phasic stimulations when compared with PGVG-treated mice (Fig. 7*C1*; two-way ANOVA, *F*_(1,17)_ = 61.5, *p <* 0.0001, drug factor; *F*_(1,17)_ = 0.58, *p =* 0.46, stimulation factor; *F*_(1,17)_ = 0.58, *p =* 0.46, interaction; *p <* 0.0001 and 0.0002, tonic and phasic, respectively; *post hoc* Bonferroni). In female mice, we only observed a significant increase in DA release following phasic stimulation (Fig. 7*D1*; two-way ANOVA, *F*_(1,22)_ = 13.4, *p =* 0.001, drug factor; *F*_(1,22)_ = 0.05, *p =* 0.83, stimulation factor; *F*_(1,22)_ = 0.47, *p =* 0.50, interaction; *p =* 0.09 and 0.01 for tonic and phasic, respectively; *post hoc* analysis). Previous investigations have shown that nicotine alters the phasic/tonic ratio of DA release (Rice and Cragg, 2004). Accordingly, we examined phasic/tonic ratios in mice exposed to GA-mix and observed that male and female mice exhibited no change in phasic/tonic ratios (*p =* 0.21 and 0.84 for males and females, respectively; Fig. 7*C2*,*D2*). Together, these data suggest that chemical flavorants enhance NAc core DA release in a manner that affects male mice at a magnitude greater than females but does not alter the phasic/tonic ratio.

To determine how GA flavorants altered nAChR-mediated DA release in the NAc core, we applied the β2* nAChR antagonist DhβE (10 μm, IC_90_) during FSCV assays (Fig. 7*E*,*F*). Prior research has shown that DhβE decreases dorsal striatal DA release by 50% (0.1 μm; Chen et al., 2019) and NAc core DA release by 80% (1 μm; Cachope et al., 2012). We exposed a separate cohort of male and female mice to PGVG and GA-mix using an identical noncontingent paradigm as described above. Following this, we examined NAc core DA release following 5 Hz and 60 Hz stimulations in the absence and presence of DhβE. Here, we detected a significant effect of DhβE (*F*_(1,35)_ = 107, *p <* 0.0001) but no significance for factors of drug/stimulation (*F*_(3,35)_ = 0.82, *p =* 0.49) or interaction (*F*_(3,35)_ = 0.85, *p =* 0.48) in male mice. We also detected a significant effect of DhβE (*F*_(1,25)_ = 197, *p <* 0.0001) but not drug/stimulation (*F*_(3,25)_ = 0.24, *p =* 0.87) or interaction (*F*_(3,25)_ = 0.47, *p =* 0.71) in female mice.

In control (PGVG treated) male mice, we observed DhβE to inhibit NAc core DA release by 59.0% and 75.8% for 5 Hz and 60 Hz stimulations, respectively (Fig. 7*E*; *p =* 0.0009 and *p <* 0.0001, respectively). Male mice exposed to GA-mix were observed to have a DhβE-induced inhibition of NAc core DA release of 49.1 and 64.4% for 5 Hz and 60 Hz stimulations, respectively (Fig. 7*E*; *p =* 0.03 and *p =* 0.002, respectively). We observed no difference in DhβE-induced changes in tonic DA release between PGVG-treated and GA-mix-treated male mice (*p >* 0.99; Fig. 7*E*). Similarly, we observed no difference in DhβE-induced changes in phasic DA release between PGVG-treated and GA-mix-treated male mice (*p >* 0.99; Fig. 7*E*). In control (PGVG treated) female mice, we observed DhβE to inhibit NAc core DA release by 73.0 and 60.0% for 5 Hz and 60 Hz stimulations, respectively (Fig. 7*F*; *p <* 0.0001). Mice exposed to GA-mix were observed to have a DhβE-induced inhibition of NAc core DA release of 66.0 and 65.0% for 5 Hz and 60 Hz stimulations, respectively (Fig. 7*F*; *p <* 0.0001). We observed no difference in DhβE-induced changes in tonic DA release between PGVG-treated and GA-mix-treated female mice (*p >* 0.99; Fig. 7*F*). Similarly, we observed no difference in DhβE-induced changes in phasic DA release between PGVG-treated and GA-mix-treated female mice (*p >* 0.99; Fig. 7*F*). Thus, in both male and female mice, exposure to GA-mix decreased DhβE-induced inhibition of NAc core DA release. This suggests that the increase in DA release by GA flavorants may not be accompanied by any change in nAChR-mediated DA release.

### Reinforcement-related behavior associated with GA flavorants correlates inversely to MHb neuronal excitability

Up to this point, all functional assays have used mice that were exposed to GA flavorants through a noncontingent drug delivery paradigm. Accordingly, this does not adequately inform us of the physiological changes that may occur with volitional drug intake. To address this, we trained a separate cohort of male and female mice to self-administer GA-mix in an EVSA paradigm (5 d acclimation, 10 d FR1, and 5 d FR3; Fig. 1*A*). Here, male mice and female mice exhibited similar numbers of FR3 active nose pokes as those exposed to GA-mix in the within-subjects design (Fig. 1*A*). At the completion of the self-administration assays, brains were extracted and used for electrophysiology assays. Brain slices containing the VTA and MHb, identical to the prior described electrophysiology assays (Figs. 4, 5, 6), were prepared. In each brain region, we targeted α6-GFP-positive dopamine neurons in the VTA and α6-GFP-positive neurons in the medial MHb. In regard to the latter, we focused on the medial MHb because of the observed changes we noted following noncontingent exposure to GA-mix. We used a stepped-current protocol to determine rheobase (minimum current to elicit an action potential) and observe the maximum number of action potentials that occur within the current steps. Here, our intent was to observe how the intrinsic excitability of these neuronal populations changed as a consequence of FR3 EVSA responding of an individual mouse (mean FR3 active nose pokes).

When we correlated our electrophysiological data from VTA pDA neurons to mean FR3 active nose pokes, we observed that neither rheobase (*r*^2^ = 0.012, *p =* 0.75) nor the maximum number of action potentials (*r*^2^ = 0.055, *p =* 0.49) correlated with mouse FR3 reinforcement-related behavior (Fig. 8*C_1,2_*). When we compared FR3 reinforcement-related behavior to medial MHb neuronal activity, we detected a significant correlation between mean FR3 active nose pokes and rheobase (Fig. 8*F_1_*; *r*^2^ = 0.523, *p =* 0.65; Fig. 8*F_2_*) as well as mean FR3 active nose pokes and maximum spikes per voltage step (*r*^2^ = 0.65, *p =* 0.0048; Fig. 8*F_1_*).

### GA flavorants alter nAChR stoichiometry

To examine flavorant-induced changes in nAChR assembly and stoichiometry, we used an *in vitro* pixel-based FRET method that has been previously validated to detect changes in nAChR stoichiometry (Srinivasan et al., 2011, 2012; Henderson et al., 2014, 2017). Here, neuroblastoma-2a cells were transiently transfected with nAChR subunits and then treated with hexyl acetate, ethyl acetate, or methylbutyl acetate separately (0.5 μm) for 24 h. Following this, NFRET methods were used to examine changes in nAChR stoichiometry.

**Figure 9.**
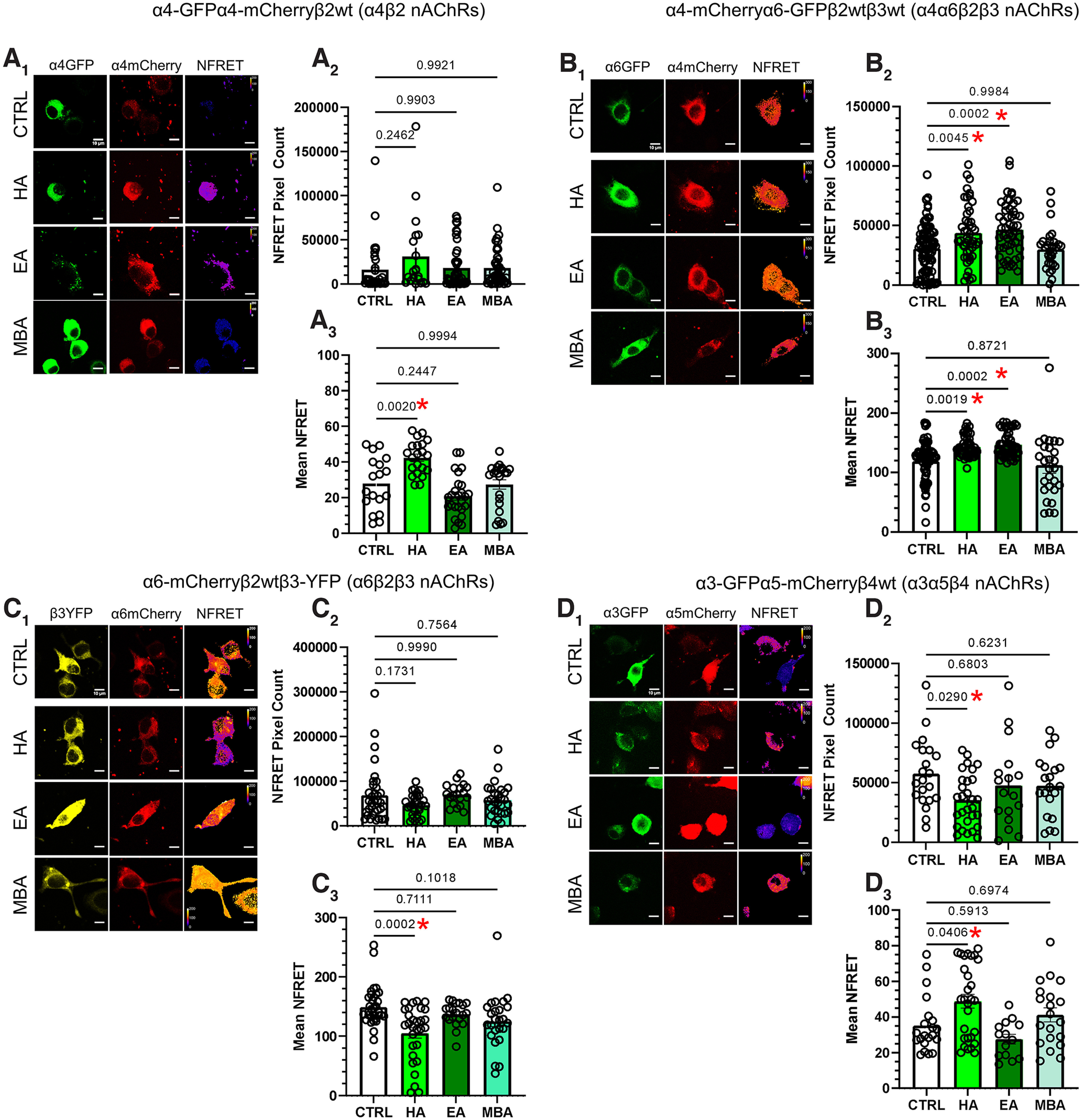
Individual GA flavorants alter nAChR stoichiometry. ***A_1_***, ***B_1_***, ***C_1_***, ***D_1_***, Representative neuroblastoma-2a cells transfected with corresponding nAChR subunits in control (CTRL)-, HA-, EA-, and MBA-treated cells. ***A_2_***, ***B_2_***, ***C_2_***, ***D_2_***, Mean NFRET pixel count for transfected cells treated 24 h with CTRL, HA, EA, or MBA. ***A_3_***, ***B_3_***, ***C_3_***, ***D_3_***, Mean NFRET of transfected cells treated 24 h with CTRL, HA, EA, or MBA. All data are mean ± SEM. One-way ANOVA with *post hoc* Bonferroni test. Dots within bars represent the values from individual cells within the designated treatment group; *n* > 30 cells per condition.

Neuroblastoma-2a cells transiently transfected with α4-mCherryα4-GFPβ2-WT nAChRs (Fig. 9*A*) exhibited a significant increase in mean NFRET in response to hexyl acetate exposure (Fig. 9*A_3_*; one-way ANOVA, *F*_(3,84)_ = 2.75, *p <* 0.0001; hexyl acetate, *p =* 0.002; ethyl acetate, *p =* 0.24; methylbutyl acetate, *p =* 0.99; *post hoc* Bonferroni), which indicates a change in nAChR stoichiometry toward low-sensitivity α4_(3)_β2_(2)_ nAChRs (Henderson et al., 2016), in agreement with our electrophysiological investigation focused on VTA GABA neurons (Fig. 4*F*). We found no flavorant-induced change in the mean pixel count of α4-GFPα4-mCherryβ2 nAChRs (Fig. 9*A_2_*; one-way ANOVA, *F*_(1,139)_ = 1.37, *p =* 0.25).

For α4α6β2β3 (α4-mCherryα6-GFPβ2-WTβ3-WT) nAChRs (Fig. 9*B*), we observed that cells exposed to hexyl acetate and ethyl acetate exhibited higher NFRET energy transfer (one-way ANOVA, *F*_(3,208)_ = 10.0, *p <* 0.0001; hexyl acetate, *p =* 0.002; ethyl acetate, *p =* 0.0002) and an increased number of NFRET pixels (one-way ANOVA, *F*_(3,211)_ = 8.73, *p <* 0.0001; hexyl acetate, *p =* 0.005; ethyl acetate, *p =* 0.0002). The first indicates that there is likely to be a shift toward a (α4)_2_(α6)_1_(β2)_2_ stoichiometry as the inclusion of more acceptor fluorophores (α4-mCherry) would exhibit higher energy. The latter indicates there is an increase in the number of assembled nAChR pentamers that include both α4 and α6 nAChR subunits. The significance of this observation is difficult to measure. Although α4α6β2 nAChRs are highly implicated in nicotine-related reward (Liu et al., 2012; Engle et al., 2013; Akers et al., 2020), the various stoichiometries that may constitute this particular subtype have not been fully characterized.

Next, we examined α6β2* (α6-mCherryβ2-WTβ3-YFP) nAChRs (Fig. 9*C*) that have been shown to have a significant difference in potency for nAChR ligands and surface expression depending on the inclusion of β3 nAChR subunits (Tumkosit et al., 2006; Kuryatov and Lindstrom, 2011; Xiao et al., 2011; Henderson et al., 2014). Here, we observed that cells treated with hexyl acetate exhibited a decrease in mean NFRET (one-way ANOVA, *F*_(3,104)_ = 6.74, *p =* 0.0003; hexyl acetate, *p =* 0.0002; *post hoc* Bonferroni). All other drug treatments resulted in a nonsignificant change in mean NFRET and pixel count (*p* > 0.05). This indicates that hexyl acetate treatment decreases the inclusion of β3 nAChR subunits to produce more low-sensitivity α6β2(non-β3) nAChRs. Finally, we examined α3α5β4 (α3-GFPα5-mCherryβ4-WT) nAChRs (Fig. 9*D*) as they have been shown to be crucial in aversion-related behaviors (Fowler et al., 2011; Frahm et al., 2011). Here, we observed that cells treated with hexyl acetate exhibited a significant decrease in NFRET pixel count (one-way ANOVA, *F*_(3,83)_ = 2.75, *p =* 0.048; *p =* 0.03, *post hoc* Bonferroni) but a significant increase in mean NFRET (one-way ANOVA, *F*_(3,80)_ = 5.33, *p =* 0.002; *p =* 0.04, *post hoc* Bonferroni). Given that α5 nAChR subunits exist as only auxiliary subunits, the increase in mean NFRET suggests that hexyl acetate-induced an increase in α3α5* nAChRs (Fig. 9*D_1–3_*).

To determine whether these chemical flavorants produce acute actions on nAChRs, we transiently transfected α4β2 nAChRs into HEK293T cells. Using a fluorescent calcium assay, we treated these cells with up to 100 μm of hexyl acetate, ethyl acetate, and methylbutyl acetate but failed to detect any nAChR response when compared with 300 μm nicotine (Fig. 9*A_1–3_*). We previously showed that another chemical flavorant, farnesol, acted as a nAChR antagonist (Avelar et al., 2019). We conducted a similar assay where increasing concentrations of the chemical flavorants were administered with nicotine to test for antagonist activity. Here, we observed that hexyl acetate, ethyl acetate, and methylbutyl acetate exhibited no efficacy as α4β2 nAChR antagonists (Fig. 9*B*). These data suggest that hexyl acetate, ethyl acetate, and methylbutyl acetate are unlikely to acutely alter nAChRs at vaping-relevant concentrations.

## Discussion

There have been several indications that ENDS users prefer flavored products (Schneller et al., 2019; Lanza et al., 2020; Leventhal et al., 2020; Jackson et al., 2021); however, there have been few reports that investigate how vaping-relevant exposures to chemical flavorants alter neurobiology. Our EVSA data showed that green apple flavor in the absence of nicotine is reinforcing to male and female mice. The fact that mice increase active nose pokes between FR1 and FR3 sessions, maintain active and inactive nose poke distinction, and exhibit extinction-related behavior when assigned vehicle (PGVG), all support the fact that the results detailed here regarding chemical flavorants are relevant to reinforcement-related behaviors. Our subsequent assays that paired EVSA and electrophysiology in the same mice suggest that stable self-administration of GA flavorants is not correlated to the intrinsic excitability of VTA pDA neurons. When we correlated individual mouse FR3 active nose pokes to VTA pDA neuron intrinsic excitability we observed a relatively flat and nonsignificant correlation (Fig. 8). However, we found that self-administration of GA flavorants correlated inversely with excitability of α6-positive medial MHb neurons. To be clear, mice that exhibited higher levels of FR3 active nose pokes exhibited lower levels of intrinsic excitability (higher rheobase, fewer action potentials). These data suggest that the amount of GA flavorant intake may be controlled by the excitability of neurons in the medial MHb. Our upregulation assays (Fig. 6) provide agreement that there is a GA-induced change in the medial MHb as we observed a GA-induced increase in α6-containing nAChRs in the medial MHb (significant for females, nonsignificant for males) but no change in the α4-containing nAChRs in the lateral MHb.

Prior reports have shown that modifications to nAChRs in the MHb can produce dramatic changes in nicotine intake. An excellent example of this was demonstrated by Fowler et al. (2011) when knockdown of the α5 nAChR subunit in the MHb resulted in the increase of the number of nicotine infusions earned at higher doses that are typically sufficient to induce aversion. This study by Fowler et al. (2011) demonstrated that knockdown of α5 nAChR subunits specifically in the MHb did not alter the rewarding effects of nicotine but abolished the inhibitory effects of higher nicotine doses. This prior work highlights the role of the MHb in aversive-related behaviors, and it clearly demonstrated that the MHb controls nicotine intake. Our investigation exhibited some similarity to this prior report as we have observed that decreased activity of these α6-containing medial MHb neurons does correlate with increased intake of GA-flavorants in our EVSA assays.

Although GA flavorants are commonly used with nicotine, the primary focus of this work was to examine how GA flavorants alter neurobiology in the absence of nicotine. It is important to highlight the fact that we observed GA flavorants produce cellular changes that are distinctly different from nicotine. Nicotine is well documented to stabilize high-sensitivity nAChRs (Kuryatov et al., 2005; Srinivasan et al., 2011; Govind et al., 2012; Fox-Loe et al., 2017; Fu et al., 2019) in a cell- and region-specific manner (Nashmi et al., 2007). This, in part, results in the fast-desensitization of VTA GABA nAChRs during repeated acute exposures to nicotine (Mansvelder et al., 2002). We observed that GA exposure increased high-sensitivity α4α6* nAChRs on VTA DA neurons (Fig. 2, males only). However, we also observed that α4β2 nAChRs on VTA GABA neurons exhibited an increase of low-sensitivity (α4)_3_(β2)_2_ nAChRs following exposure to GA (Fig. 4). This interpretation is based on our electrophysiological findings in which we observed that VTA GABA neurons exhibited decreased baseline firing and decreased potency for ACh. The decrease in VTA GBA neuron firing is likely one possible cause of the observed increase in baseline firing of VTA pDA neurons (Fig. 4). This also highlights another key difference between GA flavorants and nicotine. In prior electrophysiological investigations that used coronal brain slice preparations, long-term nicotine treatment produces a decrease in VTA DA neuron baseline firing (Nashmi et al., 2007). Here, we observed GA flavorants to produce an increase in VTA pDA neuron baseline firing. We also note that in previous reports, we found another GA flavorant, farnesol, also produced increased baseline firing of VTA DA neurons (Avelar et al., 2019), but the GA flavorant farnesene did not change VTA pDA neuron firing (Cooper et al., 2020). This highlights the fact that although there are several different chemical flavorants used in green apple flavors in ENDS, they each have distinct chemical structures and will therefore exhibit different effects.

**Figure 10.**
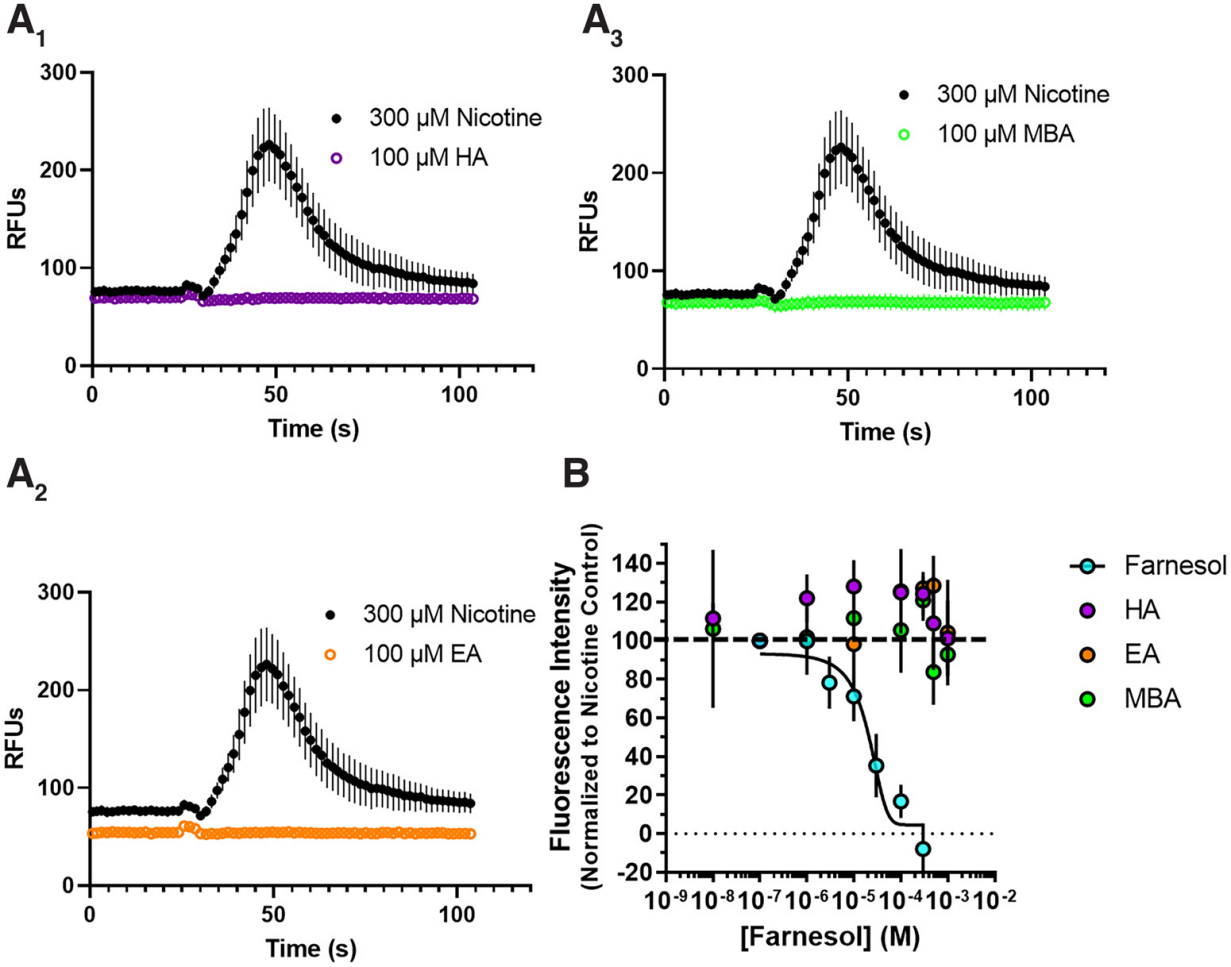
Individual GA flavorants do not act as agonists or antagonists to α4β2 nAChRs. ***A_1–3_***, HA, EA, and MBA were added to HEK293T cells transiently transfected with α4β2 nAChRs at concentrations up to 100 μm in comparison with 300 μm nicotine. HA, EA, and MBA failed to activate α4β2 nAChRs. ***B***, Increasing concentrations of HA, EA, and MBA were coapplied with 300 μm nicotine to test for potential antagonist activity. HA, EA, and MBA failed to inhibit nAChR activity at all concentrations tested. Farnesol, which has previously been shown to act as a nAChR antagonist (Avelar et al., 2019), has been shown as a comparison with HA, MBA, and EA. Data are mean ± SEM of three independent experiments (***B***).

Along this line of assessment, we noted that not all flavorants altered nAChR assembly. Hexyl acetate was the only GA flavorant to change stoichiometry of all nAChRs studied by NFRET, whereas ethyl acetate was detected only to alter α4α6* nAChRs. Methylbutyl acetate was not observed to change any nAChR stoichiometry in *in vitro* NFRET assays. This indicates that some chemical flavorants provide sufficient stimuli to self-administer; but of these flavorants, not all will alter nAChR stoichiometry or assembly. This highlights the fact that there are other mechanisms that influence intake of flavorants such as olfaction or taste. There are several follow-up investigations that need to be completed to fully investigate this. Additionally, there are several effects that deserve more precise investigations. First, we did note a potential change in α3α5* nAChRs with our *in vitro* assays. We currently lack the capability of conducting assays similar to what was done with our α4-mCherryα6-GFP mice; but using fluorescent α3 nAChR mice is a viable option for future studies. Overall, of the effects we observed, we do not suspect that GA flavorants act through acute actions on nAChRs as these chemical flavorants investigated here do no act as agonists or antagonists of nAChRs (Fig. 10). It is also important to note that our observations here show consistency. Similar to our previous work (Cooper et al., 2021), we have shown here that mice will self-administer chemical flavorants in the absence of nicotine. However, in the present report we extend the findings to show that reinforcement-related behavior to individual flavorants will be maintained on a FR3 schedule in mouse EVSA assays. EVSA remains a novel paradigm, and few groups have used vaporized nicotine in rodent self-administration paradigms (Cooper et al., 2021; Henderson and Cooper, 2021; Lallai et al., 2021).

Altogether, our data show that GA flavorant mixtures, consistent with current-market e-liquids, can have an impact on reinforcement-related behaviors. This occurs in the absence of nicotine and accompanied by changes in nAChR number and stoichiometry. Finally, our data suggest that intake of GA flavorants is correlated to excitability of MHb neurons (inversely) and not VTA DA neurons. These data may provide mechanistic details on why ENDS users of all ages may prefer certain flavored products.
